# Identifying the Key Factors Affecting Warning Message Dissemination in VANET Real Urban Scenarios

**DOI:** 10.3390/s130405220

**Published:** 2013-04-19

**Authors:** Manuel Fogue, Piedad Garrido, Francisco J. Martinez, Juan-Carlos Cano, Carlos T. Calafate, Pietro Manzoni

**Affiliations:** 1 DIIS, University of Zaragoza, Ciudad Escolar s/n, Teruel 44003, Spain; E-Mails: mfogue@unizar.es (M.F.); piedad@unizar.es (P.G.); 2 DISCA, Universitat Politècnica de València, Camino de Vera s/n, Valencia 46022, Spain; E-Mails: jucano@disca.upv.es (J.-C.C.); calafate@disca.upv.es (C.T.C.); pmanzoni@disca.upv.es (P.M.)

**Keywords:** vehicular *Ad Hoc* networks, performance evaluation, inter-vehicle communication, 2*^k^* factorial analysis, real roadmaps, traffic safety

## Abstract

In recent years, new architectures and technologies have been proposed for Vehicular *Ad Hoc* networks (VANETs). Due to the cost and complexity of deploying such networks, most of these proposals rely on simulation. However, we find that most of the experiments made to validate these proposals tend to overlook the most important and representative factors. Moreover, the scenarios simulated tend to be very simplistic (highways or Manhattan-based layouts), which could seriously affect the validity of the obtained results. In this paper, we present a statistical analysis based on the 2*^k^* factorial methodology to determine the most representative factors affecting traffic safety applications under real roadmaps. Our purpose is to determine which are the key factors affecting Warning Message Dissemination in order to concentrate research tests on such parameters, thus avoiding unnecessary simulations and reducing the amount of simulation time required. Simulation results show that the key factors affecting warning messages delivery are the density of vehicles and the roadmap used. Based on this statistical analysis, we consider that VANET researchers must evaluate the benefits of their proposals using different vehicle densities and city scenarios, to obtain a broad perspective on the effectiveness of their solution. Finally, since city maps can be quite heterogeneous, we propose a roadmap profile classification to further reduce the number of cities evaluated.

## 1. Introduction

Vehicular *Ad Hoc* networks (VANETs) are wireless networks that do not require any fixed infrastructure. These networks are considered essential for cooperative driving among cars on the road. The development of VANETs is backed by strong economical interests since vehicle-to-vehicle (V2V) communication allows the sharing of wireless channels for mobile applications, improving route planning, controlling traffic congestion, improving traffic safety, and providing entertainment [1,2]. Most of these applications depend on services to disseminate warning messages, which are alert messages sent by a vehicle to warn other vehicles of any potential danger. In the coming future, vehicles will not only distribute information about themselves and their environment using warning messages, but also communicate with other vehicles and the infrastructure via multihop wireless communications [3].

Deploying and testing VANETs involves high cost and intensive labor, being prohibitive in most cases. Hence, simulation is a useful alternative prior to actual implementation [4]. Moreover, VANET simulations must account for some specific characteristics found in vehicular environments. For instance, VANET simulations often involve large and heterogeneous scenarios. Traditional mobile systems also present a large number of parameters potentially affecting their performance, thus increasing considerably the simulation time required to correctly evaluate any proposal in a wide variety of scenarios. In recent years, new architectures and technologies have been proposed for VANETs, thanks to the use of simulation. However, the experiments to validate these proposals tend to overlook the most important and representative factors. Moreover, the scenarios simulated tend to be very simplistic (highways or Manhattan-based layouts), and most of them use the 802.11g standard, already implemented in most simulators, instead of using the 802.11p [5] which is going to be used for inter-vehicular communication. Thus, we find that different proposals in the VANET field lack generality, being uncertain whether they will perform adequately in a real VANET environment.

In this paper, we present a statistical analysis based on the 2*^k^* factorial methodology [6] to determine the most representative factors that govern the warning message dissemination performance in 802.11p-based VANETs. The aim of this methodology is to reduce the simulation time required to analyze the performance of a given VANET system, since it allows researchers to focus on the key factors affecting their proposals.

We start our analysis by selecting the following nine factors that have been widely used in the literature: (i) the number of warning mode vehicles; (ii) the density of vehicles; (iii) the channel bandwidth; (iv) the broadcast scheme; (v) the message priority; (vi) the periodicity of messages; as well as (vii) the mobility model used; (viii) the radio propagation model; and (ix) the simulated roadmap. In a factorial design strategy, all factors are varied together (as opposed to one-at-time). So, a key advantage of this methodology is that it allows researchers to find out not only the most representative factors, but also the possible interactions and interdependencies among them.

Based on the aforementioned statistical analysis, we present a city profile classification, since the analysis indicates that VANET researchers must carefully evaluate the benefits of their proposals using different vehicle densities and roadmap scenarios, in order to make their conclusions more representative and closer to reality.

This paper is organized as follows. Section 2 describes related work on the factors commonly studied in VANETs, and the use of 2*^k^* factorial analyses in wireless networks. Section 3 presents the 2*^k^* factorial analysis fundamentals. Section 4 describes the main factors of interest in VANET research. In Section 5 we determine the key factors in VANET simulation using the 2*^k^* factorial analysis; based on the simulation results, we then provide some guidelines for future research. In Section 6 we propose and evaluate a roadmap profile classification that allows researchers to better assess their proposals. Finally, Section 7 concludes this paper.

## 2. Related Work

In this section we present some of the most representative works regarding: (i) the factors commonly studied in VANETs; and (ii) the use of 2*^k^* factorial analyses in wireless networks.

### 2.1. Factors Commonly Studied in VANETs

Most currently available VANET research works rely on simulation. However, we find that most of the experiments made to validate these proposals tend to overlook the most important and representative factors.

Zuo *et al.* [7] proposed the vehicle-node density parameter to improve the performance of both AODV and OLSR routing protocols under two typical mobile models in VANET. Simulation results showed the performance improvements of routing protocols when increasing the node density around the receiver. In this work, they varied the density of vehicles and the mobility models, while maintaining unaltered other parameters such as the simulation area, the transmission range, the packet size, and the radio propagation model.

Giordano *et al.* [8] focused on the accuracy of urban propagation models and their impact on vehicular protocol results. They compared the Two Ray model and the Corner model in a city scenario. Moreover, they identified a number of factors that undermine the validity of the Two Ray model, for example, the presence of buildings causing propagation disruption and the heavy weight border effects that incorrectly compensate for the presence of hidden terminals in the networks. In this work, authors varied the transmission range, the map size and the radio propagation model, while maintaining unaltered other parameters such as the density of vehicles, the packet size, *etc*.

Khorashadi *et al.* [9] looked at the result of tuning transmission power and its effect on UDP throughput in VANETs. Results showed that the major mitigating factor in VANETs is the number of hops between the source and the destination. They assessed that increasing the transmission range results in decreasing the number of hops between source and destination effectively increasing throughput. Authors also found that the effect of vehicle densities is only important at lower transmission ranges to provide the required connectivity.

Regarding warning message dissemination, Cenerario *et al.* [10] described in detail a vehicular dissemination protocol that allows sharing information such as available parking spaces, accidents or obstacles in the road, *etc.*, by using vehicle-to-vehicle communications. In this work, authors varied some factors such as the density of vehicles and the vehicle's speed, while maintaining unaltered other parameters such as the transmission range, the map size, the radio propagation model, the simulated roadmap, *etc.* Sahoo *et al.* [11] proposed an IEEE-802.11-based multi-hop broadcast protocol to address the issue of warning message dissemination in VANETs. The protocol adopts a binary-partition-based approach to repetitively divide the area inside the transmission range to obtain the furthest possible segment. In this work, authors varied some factors such as the density of vehicles and the vehicles' speed, while maintaining unaltered other parameters such as the periodicity of messages, the radio propagation model, the transmission range, *etc*.

The effect of obstacles in warning message dissemination has also been addressed by some works. Costa *et al.* [12] presented an approach where a message propagation function encodes information about target areas and preferred routes for the message dissemination. Selecting different functions produces different routing protocols accounting for connected and disconnected situations between vehicles. These protocols show a remarkable performance in simple grid-like scenarios with low and high density of vehicles, but real maps are not used in their simulations. Viriyasitavat *et al.* [13] proposed the UV-CAST (Urban Vehicular broadCAST) protocol, which allows reducing the broadcast storm problem while solving disconnected network problems in urban VANETs. However, the density of vehicles studied is relatively low, and the authors did not study its performance when there are more than 50 vehicles per km^2^. Liu and Chigan [14] proposed the RPB-MD protocol, a message dissemination approach with a relative position based (RPB) addressing model that allows defining the intended receivers in the zone of relevance. Simulation results show high delivery ratio and low data overhead; however, the scenario used is a single bidirectional highway, and the Radio Propagation Model selected is the deterministic Two-Ray Ground.

To the best of our knowledge, there is no research work that formally identifies the factors that significantly affect the performance of warning message dissemination systems for VANETs in real roadmaps. Hence, we consider that the contributions made in this paper offer significant guidance to the research community in this area.

#### 2.2. 2^k^ Factorial Analysis in Wireless Networks

In the networking literature we can find several works that adopted the 2*^k^* factorial approach to discriminate among the many available parameters so as to determine the most relevant ones.

Gupta *et al.* [15] studied Distributed Network Control Systems (D-NCS), a network structure and components that are capable of integrating sensors, actuators, communication, and control algorithms to suit real-time applications. Standard statistical approaches, such as 2*^k^* factorial experiment design, analysis of variance, and hypothesis testing, were used to study and estimate the effect of each factor on the system performance.

Liu *et al.* [16] studied the use of multipath routes to improve throughput, end-to-end delay, and the reliability of data transport in Wireless Sensor Networks (WSNs). They reported the results of a series of simulations based on a factorial experimental design. Results showed that both the congestion window size and the retry limit are key factors. Vaz de Melo *et al.* [17] studied how different WSNs can cooperate in order to reduce the total energy consumption. Simulation results revealed that different densities and data collecting rates among WSNs, the routing algorithm, and the path loss exponent had a major impact in the establishment of cooperation. The initial assessment of the impact of these factors was made through a 2*^k^* factorial experimental analysis.

Perkins *et al.* [18] studied and quantified the effects of various factors and their two-way interactions on the overall performance of MANETs. Using 2*^k^* factorial experimental design, they isolated and quantified the effects of five factors: (i) node speed; (ii) pause-time; (iii) network size; (iv) number of traffic sources; and (v) type of routing. They evaluated the impact that these factors have over the throughput, routing overhead, and power consumption. In [19], they investigated the impact of some characteristics on the performance of TCP in MANETs. Moreover, a factorial design experiment was conducted to quantify the effects and interactions that node speed and node pause time have over the TCP throughput.

Although the use of standard statistical approaches such as the 2*^k^* factorial analysis is found in many other fields, it is not so frequently used in *Ad Hoc* network communications. Specifically, the 2*^k^* factorial approach has been adopted to discriminate among the many available parameters so as to determine the most relevant ones. As the number of different parameters in vehicular communications is very high, we consider that this method can also be applied in VANETs [20]. As shown in Section 5.1, the two extreme values used in our 2*^k^* factorial analysis are chosen among representative extreme values, within the bounds of applicability and technical feasibility. Additionally, in Sections 5.2-5.4 we confirmed the outcome of the 2*^k^* factorial analysis by performing a sensibility analysis when varying the values of the key factors in simulations.

## 3. The 2*^k^* Factorial Analysis

VANET simulations often involve large and heterogeneous scenarios. The number of possible factors and their values, or levels, can be very large. In this section, we will explain how the 2*^k^* factorial analysis [6] can be used to determine the most relevant factors that govern a system's performance.

The use of 2*^k^* factorial is important for several reasons: (i) to reduce the overall number of simulations needed; (ii) to evaluate the relationship between different factors; and (iii) to reduce the amount of simulation time required. The basic approach of this method is based on selecting a set of *k* parameters and determining 2 extreme levels (tagged with —1 and 1). An experiment is run for all the 2*^k^* possible combinations of the parameters. From each experiment, we can also extract the $\left(\begin{array}{c} k\\ 2 \end{array}\right)$ two-factor interactions, the $\left(\begin{array}{c} k\\ 3 \end{array}\right)$ three-factor interactions, and so on.

For example, suppose that we have proposed a Warning Message Dissemination system, and that we want to study the impact of the density of vehicles (factor A) and the speed of these vehicles (factor B) on the warning notification time, *i.e.*, the time required by normal vehicles to receive a warning message sent by a warning mode vehicle.

If we make a 2^2^ factorial analysis, we can find out the impact of each factor (density of vehicles and speed), and their combination, in the studied metric (warning notification time). Table 1 shows the different experiments defined by the 2^2^ design, and Table 2 shows the results obtained after the simulations.

Let us define two variables *x_A_* and *x_B_* as presented in Equations (1) and (2):

(1) $$x_{A}=\left\{\begin{array}{ccc} -1 & \text{if} & \text{density of vehicles}=25\\ 1 & \text{if} & \text{density of vehicles}=150 \end{array}\right\}$$

(2) $$x_{B}=\left\{\begin{array}{ccc} -1 & \text{if} & \text{speed}=10\;km/h\\ 1 & \text{if} & \text{speed}=80\;km/h \end{array}\right\}$$

The warning notification time (*y*) can be regressed on *x_A_* and *x_B_* using a nonlinear regression model of the form:

(3) $$\text{y}=q_{0}+q_{A}x_{A}+q_{B}x_{B}+q_{AB}x_{A}x_{B}$$

Substituting the four observations in the model, we get the following four equations:

(4) $$1=q_{0}-q_{A}-q_{B}+q_{AB}$$

(5) $$0.5=q_{0}+q_{A}-q_{B}-q_{AB}$$

(6) $$0.8=q_{0}-q_{A}+q_{B}+q_{AB}$$

(7) $$0.4=q_{0}+q_{A}+q_{B}+q_{AB}$$

These equations can be solved uniquely for the four unknowns. The regression equation is:

(8) $$\text{y}=0.675-0.225x_{A}-0.075x_{B}+0.025x_{A}x_{B}$$

The result is interpreted as follows: the mean warning notification time is 0.675 s, the effect of the density of vehicles is -0.225 s, the effect of the speed of the vehicles is -0.075 s, and the interaction between speed and density of vehicles accounts for 0.025 s.

In a 2*^k^* factorial analysis, by using the sign table method, we can get the results and detect variations that depend on the combination of factors. For a 2^2^ design, the effects can be computed easily by preparing a 4 × 4 sign matrix as shown in Table 3. The first column of the matrix is labeled *I*, and all its elements are equal to 1. The next two columns, titled *A* and *B*, contain basically all possible combinations of –1 and 1. The fourth column, labeled *AB*, is the product of the entries in columns *A* and *B*. The four observations are listed in a column vector next to this matrix. The column vector is labeled *y* and consists of the results corresponding to the factor levels listed under columns *A* and *B*. The next step is to multiply the entries in column *I* by those in column *y* and put their sum under column *I*. The entries in column *A* are now multiplied by those in column *y* and the sum is entered under column *A*. This operation of column multiplication is repeated for the remaining two columns of the matrix. The sums under each column are divided by 4 to give the corresponding coefficients of the regression model.

The importance of a factor depends on the proportion of the metric total variation explained by the factor. The total variation of *y* is also known as Sum of Squares Total (SST), which can be calculated as follows:

(9) $$\text{Total variation of y}=\text{SST}=\sum_{i=1}^{2^{2}}{\left(y_{i}-\bar{y}\right)}^{2}$$

where *y̅* denotes the mean of the responses from all four experiments. For a 2^2^ design, the variation can be divided into three parts:

(10) $$\text{SST}=2^{2}q_{A}^{2}+2^{2}q_{B}^{2}+2^{2}q_{AB}^{2}$$

These parts can be expressed as a fraction; for example:

(11) $$\text{Fraction of variation explained by A}=\frac{\text{SSA}}{\text{SST}}=\frac{2^{2}q_{A}^{2}}{\text{SST}}$$

Hence, we can indicate the percentage of variation of each studied metric explained by each factor. The more percentage of variation, the more impact this factor has in the measured metric. In our example, we found that the density of vehicles accounts for 89.01% (*i.e.*, $\frac{2^{2}\times {\left(-0.225\right)}^{2}}{0.2275}$) of the total variation of the warning notification time, the speed of the vehicles accounts for 9.89% (*i.e.*, $\frac{2^{2}\times {\left(-0.075\right)}^{2}}{0.2275}$), and their combination accounts for the remaining 1.10% (*i.e.*, $\frac{2^{2}\times 0.025^{2}}{0.2275}$). Therefore, in our selected example the density of vehicles is the most important factor that affects the warning notification time.

The outcome of the 2*^k^* factorial analysis allows us in sorting out factors in the order of impact. At the beginning of any performance study, the number of factors and their levels could usually be large. A full factorial design with such a large number of factors and levels may not be the best use of available effort. The first step should be to reduce the number of factors and to choose those factors that have a significant impact on performance.

## 4. Factors to Study in VANETs

Some previous works have studied the most important factors in MANETs. Nevertheless, VANETs have special characteristics that make them different from MANETs. Hence, more research is required in order to identify the key factors that impact their performance. In this section we identify and describe the most important factors associated with VANET Warning Message Dissemination.

### 4.1. Number of Warning Vehicles

In traffic safety applications, vehicles may send safety messages to other vehicles in order to prevent collisions or to ask for emergency services. We consider that vehicles may operate in warning or normal mode. Warning mode vehicles inform other vehicles about their abnormal status by sending warning messages periodically. Normal mode vehicles participate in the diffusion of these warning packets and, periodically, they also send *beacons* with information about themselves, such as their position and speed.

This factor is important since the more vehicles are in the warning mode, the more network traffic, thus increasing redundant rebroadcasts that provoke heavy contention and long-lasting collisions.

### 4.2. Density of Vehicles

In VANETs, the density of vehicles can be particularly high, which usually causes that VANET simulations require quite a long time to finish. Moreover, many network simulators do not scale well, and so simulating VANETs with high density of vehicles consumes a significant amount of time and resources.

As shown in previous works [21,22], this factor seems to be important to measure Warning Message Dissemination performance in VANET scenarios. In fact, some authors have defined new compound factors derived from the density of vehicles (e.g., Jiang *et al.* [23] defined the concept of communication density as the product of vehicle density, messaging rate and transmission range).

### 4.3. Channel Bandwidth

In radio communications, bandwidth is the width of the frequency band used to transmit the data. Channel spacing is a term used in radio frequency planning that describes the frequency difference between adjacent allocations in a frequency plan.

Wireless technologies such as the IEEE 802.11p Wireless Access for Vehicular Environment (WAVE) [24] enable peer-to-peer mobile communication among vehicles (V2V) and communication between vehicles and the infrastructure (V2I), and are expected to be widely adopted by the car industry in the next years. The 802.11p standard supports 10 MHz and 20 MHz bandwidths. Using a 10 MHz bandwidth, the supported data rates are 3, 4.5, 6, 9, 12, 18, 24, and 27 Mbps, depending on the modulation and coding scheme considered.

In vehicular safety communications, the efficiency of channel usage is important in managing the broadcast transmissions. The efficient channel usage helps to reduce the overall interference level and in turn impacts on the broadcast reception performance [25].

Since vehicular information delivery systems support applications such as cooperative driving among cars on the road, traffic safety, or infotainment applications, we think that channel bandwidth requirements could change based on the selected application. For the specific case of Warning Message Dissemination mechanisms, the overall capacity of the channel can affect the effectiveness of warning dissemination schemes if the density of potential transmitters is high.

### 4.4. Broadcast Scheme

Another important factor in Warning Message Dissemination in VANETs is the selected broadcast scheme [26]. In VANETs, intermediate vehicles act as relays to support end-to-end vehicular communications. For applications such as route planning, traffic congestion control, and traffic safety, flooding of broadcast messages commonly occurs. However, flooding results in many redundant rebroadcasts, heavy channel contention, and long-lasting message collisions (usually known as the broadcast storm problem).

Over the years, several schemes have been proposed to address the broadcast storm problem in wireless networks. In [27] we can find some of the most interesting approaches, which are the following: (i) the counter-based scheme, which uses a counter to keep track of the number of times the broadcast message is received in order to decide whether to inhibit the rebroadcast; (ii) the distance-based scheme, in which the relative distance between vehicles is used to decide whether to rebroadcast or not; (iii) the location-based scheme, which is very similar to the distance-based scheme, though requiring more precise locations for the broadcasting vehicles to achieve an accurate geometrical estimation of the additional coverage of a rebroadcast; and (iv) the cluster-based scheme, where vehicles are grouped in clusters, and only one member of each cluster (the cluster head) can rebroadcast the warning messages. The *weighted p-persistence*, the *slotted 1-persistence*, and the *slotted p-persistence* techniques presented in [28] are some of the few rebroadcast schemes proposed for VANETs. These three probabilistic and timer-based broadcast suppression techniques can mitigate the severity of the broadcast storms by allowing nodes with higher priority to access the channel as quickly as possible, but their ability to avoid storms is limited, since they are specifically designed for being used in highway scenarios. The Last One (TLO) scheme [29] tries to reduce the broadcast storm problem by finding the most distant vehicle from the warning message sender, so that this vehicle will be the only one allowed to retransmit the message. This scheme does not take into account the effect of obstacles (e.g., buildings) in urban radio signal propagation. More recently, we proposed a scheme called enhanced Message Dissemination based on Roadmaps (eMDR) [21], which uses location and roadmap information to facilitate an efficient dissemination of warning messages in 802.11p-based VANETs.

It is easily noticeable that most existing solutions to the broadcast storm problem were only evaluated in obstacle-free environments, which are not comparable to real urban scenarios where plenty of obstacles can interfere with the signal, creating blind areas where vehicles will not receive the warning message unless intermediate forwarding nodes help to overpass the obstacle. In our experiments, we use both the location-based scheme and our eMDR scheme to assess the relevance of the broadcast scheme adopted.

### 4.5. Message Priority

The 802.11p MAC layer is based on the IEEE 802.11e Enhanced Distributed Channel Access (EDCA), and Quality of Service (QoS) extensions. Therefore, application messages are categorized into different Access Classes (ACs), where AC0 has the lowest and AC3 the highest priority.

In our experiments, *warning messages* (which contain information about abnormal situations such as accidents) have always the highest priority (AC3) at the MAC layer, while *beacons* (containing information such as vehicles' positions and speeds), which are not propagated by other vehicles, change their priority from the lowest (AC0) to the highest (AC3) priority in the 2*^k^* factorial analysis.

### 4.6. Message Periodicity

As mentioned previously, warning mode vehicles inform other vehicles about their status by sending warning messages periodically. Normal mode vehicles participate in the diffusion of these warning packets and, moreover, they also send periodic *beacons* with information such as their positions, speed, *etc.*

Similarly to the number of warning vehicles, the more warning messages are sent at the same time, the more redundant rebroadcasts, channel contention, and message collisions there will be. Thus, message periodicity seems to be an important factor that offers a trade-off between performance and overhead.

### 4.7. Mobility Model

One of the challenges posed by the study of VANETs is the definition of a vehicular mobility model [30] providing an accurate and realistic vehicular mobility description at both macroscopic and microscopic levels [31]. To perform realistic simulations, it is especially important that the chosen mobility generator is able to obtain a detailed microscopic traffic simulation by importing network topologies from real maps. Our mobility simulations are performed with SUMO [32], an open source traffic simulation package that has interesting microscopic traffic capabilities, such as collision free vehicle movement, multi-lane streets with lane changing, junction-based right-of-way rules, traffic lights, *etc.* SUMO can also import roadmaps directly from map databases such as OpenStreetMap [33] and TIGER [34].

Our mobility simulations account for areas with different vehicle densities. In a real town, traffic is not uniformly distributed; there are downtowns or points of interest that may attract vehicles. Hence, we include the ideas presented in the *Downtown Model* [35] to add points of attraction in realistic roadmaps.

To generate the movements for the simulated vehicles, we used two different mobility models available in SUMO: (i) the Krauss mobility model [36] with some modifications to allow multi-lane behavior [37]; and (ii) the Wagner mobility model [38]. The Krauss model is based on collision avoidance among vehicles by adjusting the speed of a vehicle to the speed of its predecessor using the following formula:

(12) $$v\left(t+1\right)=v_{1}\left(t\right)+\frac{g\left(t\right)-v_{1}\left(t\right)\tau }{\tau +1}+\eta \left(t\right)$$

where *v* represents the speed of the vehicle in m/s, *t* represents the period of time in seconds, *v*_1_ is the speed of the leading vehicle in m/s, *g* is the gap to the leading vehicle in meters, *τ* is the driver's reaction time (set to 1 second in our simulations) and *η* is a random numeric variable with a value between 0 and 1.

The Wagner model, unlike most driving models that assume an instantaneous or even delayed reaction of the driver to the surrounding situation, considers two important features of human driving and of human actions in general. Firstly, humans usually plan ahead, and secondly, the type of control that humans apply is not continuous, but discrete in time: they act only at certain moments in time. These specific moments are known as action-points.

### 4.8. Radio Propagation Model

We observe that the most widely used simulators such as ns-2, Glomosim, QualNet and OPNET do not include a Radio Propagation Model (RPM) that offers enough accuracy for vehicular environments [39]. In particular, the physical obstacles present in urban environments (mostly buildings) are not taken into account, which is overly optimistic. For example, the commonly used Two Ray Ground (TRG) radio propagation model ignores effects such as Radio Frequency (RF) attenuation due to buildings and other obstacles, meaning that an alternative model must be introduced. However, for 802.11p-based VANETs, the received signal will largely depend on both the distance between the sender and the receiver, and the presence of obstacles.

In the 2*^k^* factorial analysis, we use both the well-known deterministic TRG and the probabilistic Real Attenuation and Visibility Model (RAV) [4], a realistic RPM specifically designed for IEEE 802.11p-based VANETs that increases the level of realism of phenomena occurring at the physical layer, thereby allowing researchers to obtain more accurate and meaningful results [39].

**Figure 1.**
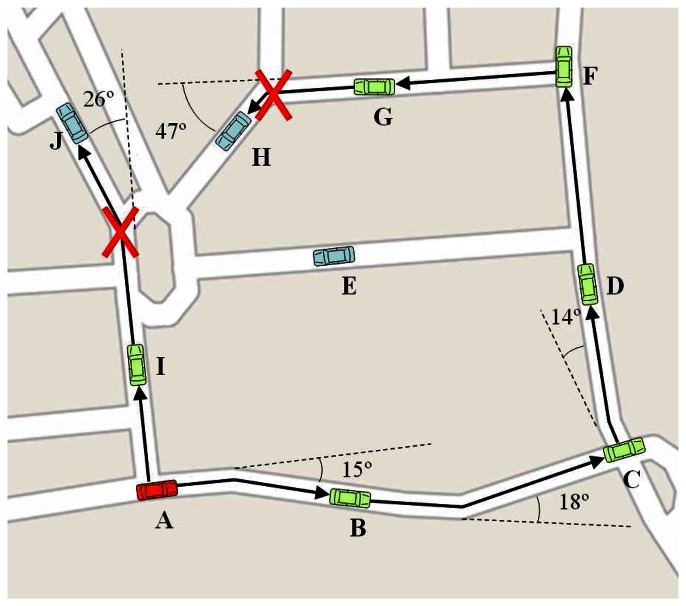
RAV visibility scheme: example scenario.

Figure 1 shows an example of the visibility scheme used in RAV, where vehicle (A) is trying to disseminate a message. In that case, and assuming that any vehicle receiving a message will rebroadcast it the first time, the result will be that some vehicles (B, C, D, F, G, and I) receive the message, while the others (E, H, and J) will never be reached by such message.

### 4.9. Roadmap

The roadmap (road topology) is an important factor accounting for mobility in simulations, since the topology constrains cars' movements. Roughly described, an urban topology is a graph where vertices and edges represent, respectively, junction and road elements. Simulated road topologies can be generated *Ad Hoc* by users, randomly by applications, or obtained from real roadmap databases. Using complex layouts implies more computational time, but the results obtained are closer to the real ones [21]. Typical simulation topologies used are highway scenarios (the simplest layout, without junctions) and Manhattan-style street grids (with streets arranged orthogonally). These approaches are simple and easy to implement in a simulator. However, layouts obtained from real urban scenarios are rarely used, although they should be chosen to ensure that the results obtained are likely to be similar in realistic environments.

**Figure 2.**
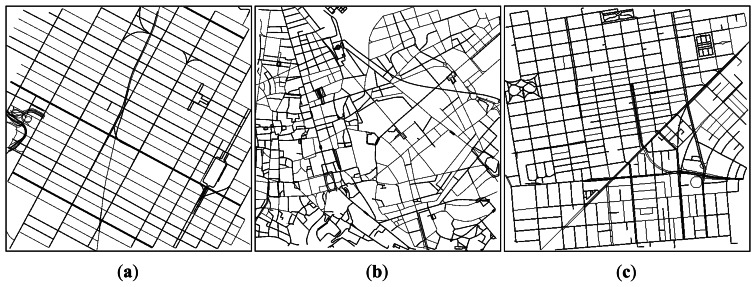
Scenarios used in our simulations as street graphs in SUMO: (**a**) fragment of the city of New York (USA); (**b**) fragment of the city of Rome (Italy); and (**c**) fragment of the city of San Francisco.

Our simulation scenarios used in the 2*^k^* factorial analysis are based on two different real roadmaps, which were obtained from real cities using OpenStreetMap. The two locations represent environments with different street densities and average street lengths. The chosen scenarios were the South part of the Manhattan Island from the city of New York (USA), and the area located at the North of the Colosseum in the city of Rome (Italy). The fragments selected have an extension of 4 km^2^ (2 km × 2 km). Figure 2 depicts the street layouts used. As shown, the fragment from New York presents the longest streets, arranged in a Manhattan-grid style. The city of Rome represents the opposite situation, with short streets in a highly irregular layout. The third fragment was extracted from the city of San Francisco, and the results of its simulation are presented in Section 5.4.

## 5. Simulation Results

Simulation results presented in this paper were obtained using the ns-2 simulator [40]. We modified the simulator to follow the upcoming WAVE standard closely (all these improvements and modifications of the simulator are publicly available at http://www.grc.upv.es/software/), extending it to implement IEEE 802.11p [5]. Mobility is performed with CityMob for Roadmaps (C4R) [41], a mobility generator that can import maps directly from OpenStreetMap.

In our study, each simulation lasted for 120 s. In order to achieve a stable state before gathering data traffic, we only started to collect data after the first 60 s. All results represent an average over thirty executions with different random scenarios, presenting all of them a maximum error of 10% with a degree of confidence of 90%. We evaluated the following performance metrics: (i) the warning notification time; (ii) the percentage of blind vehicles; and (iii) the number of packets received per vehicle. The warning notification time is the time required by normal vehicles to receive a warning message sent by a warning mode vehicle. The percentage of blind vehicles is the percentage of vehicles that does not receive the warning messages sent by the warning mode vehicles. These vehicles can remain blind because of their positions, due to collisions, or due to signal propagation limitations. Table 4 shows the parameters used for the simulations. The downtown probability and the downtown attraction are the probability that a vehicle is within downtown and the probability that a vehicle travels into downtown area, respectively.

### 5.1. Results of the 2^k^ Factorial Analysis

In this section, we use the 2*^k^* factorial analysis [6] to determine the most relevant factors that govern Warning Message Dissemination performance. We consider 9 factors, previously presented in Section 4. They are listed in Table 5. We tag each of the factors with A, B, C, …, I accordingly, as stated in the table. Thereafter, we specify two representative and basically opposite scenarios, which are described by two different levels, *i.e.*, Level –1 and Level 1. Each level provides different parameter values to define the scenario.

After having executed the 2*^k^* factorial analysis, Table 6 indicates the percentage of variation of each studied metric explained by each factor. The more the percentage of variation, the more impact this factor has in the measured metric.

The results of our 2*^k^* factorial analysis show that:

- The average time required to complete the propagation process is largely affected by the RPM used (H), the simulated roadmap (I), the combination of the density and the mobility model (BG), and the combination of the density and the RPM used (BH).
- The average number of blind vehicles is largely affected by the density of vehicles (B), the RPM used (H), the simulated roadmap (I), and the combination of the density and the RPM used (BH).
- The average number of packets received per vehicle is largely affected by the density of vehicles (B), the RPM used (H), and the simulated roadmap (I).

Based on the above outcome, we can state that the key factors to be accounted for when studying warning dissemination systems are the density of vehicles, the radio propagation model, and the simulated roadmap. We now perform a detailed study to evaluate the impact of the most representative factors one by one.

### 5.2. Evaluating the Impact of the Radio Propagation Model

**Figure 3.**
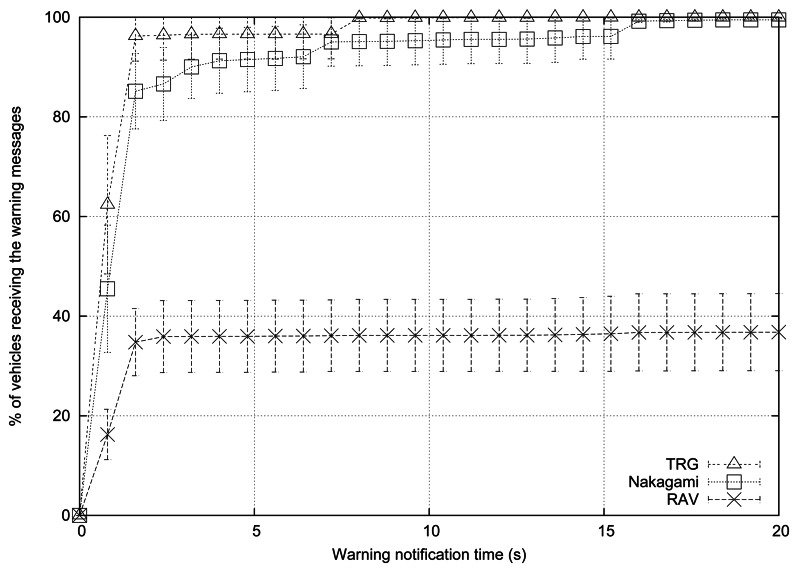
Cumulative histogram for the time evolution of disseminated warning messages when varying the RPM used.

Figure 3 shows the simulation results when varying the number of vehicles. We selected the TwoRay Ground, the Nakagami fading, and the RAV models. Table 4 shows some of the parameters used for the simulations; the rest of parameters are the following: the roadmap used is Rome, vehicles follow the Krauss mobility model, there are 3 warning mode vehicles, the periodicity of messages is 1 message per second, normal message priority is AC0, the broadcast scheme applied is eMDR, and the channel bandwidth is 6 Mbps.

According to the 2*^k^* factorial analysis, the results show that the warning notification time is highly affected by the RPM used. When using the TRG model, information reaches 30% of the vehicles in less than 1 s, and propagation is completed in less than 8 s. When using the RAV model, the system needs 2 s to reach 30% of the vehicles, although the propagation process was completed in only 2.5 s.

Table 7 shows the percentage of blind vehicles and the number of packets received per vehicle when varying the RPM. As shown, the behavior in terms of percentage of blind vehicles and the number of packets received also highly depends on this factor. In fact, when using TRG and Nakagami fading models, there are practically no blind vehicles, while we find 60.92% of blind vehicles when using RAV. Therefore, when the model is more realistic, more time is needed to reach the same percentage of vehicles, and thus the percentage of blind vehicles increases. This occurs because both TRG and Nakagami models are really optimistic, and they do not account for the presence of obstacles in signal propagation. Moreover, the average number of packets received per vehicle highly differs depending on the model (see Table 7). The number of packets received decreases considerably for RAV since signal propagation encounters more restrictions.

**Figure 4.**
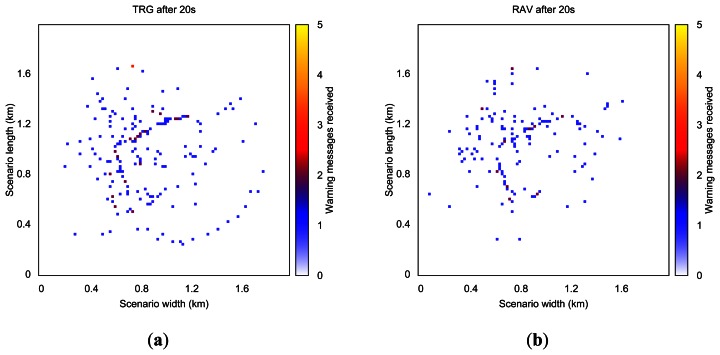
Evolution of the warning message dissemination process in the Rome scenario after 20 s, when using (**a**) the TwoRay Ground and (**b**) the RAV model.

In order to better understand the warning dissemination process, Figure 4 offers a heat map of the number of messages received in one of our simulations at different time instants. Each heat map was obtained by splitting the Rome scenario in a 100 × 100 grid, meaning that each cell depicted represents 400 m^2^ (20 m × 20 m).

Figure 4 shows the number of warning messages received in each area when using TRG and RAV radio propagation models, respectively. White areas indicate that no messages were received during the simulation (blind zones and buildings), whereas yellow areas represent locations where 5 or more messages were received. Yellow areas indicate more messages received and blue areas represent fewer messages.

When using the TRG model the dissemination process is able to reach a wider area of the scenario since the signal encounters no restrictions except the maximum transmission range. The results show that using a more realistic model tends to reduce protocol performance, allowing us to better understand the impact of buildings and obstacles along the road on car-to-car communications. Although the RAV model yields poorer performance results than TRG, it is in fact a more realistic radio propagation model, which should be considered in VANET simulations.

### 5.3. Evaluating the Impact of the Density of Vehicles

**Figure 5.**
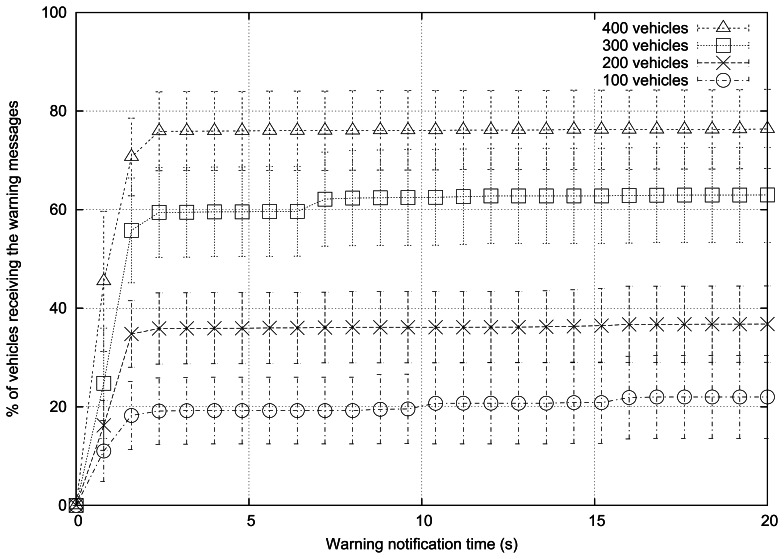
Warning notification time when varying the density of vehicles.

Figure 5 shows the simulation results when varying the number of vehicles. We selected 100, 200, 300, and 400 vehicles (*i.e.*, 25, 50, 75, and 100 vehicles/km^2^). Table 4 shows some of the parameters used for the simulations; the rest of parameters are the following: the roadmap used is Rome, the radio propagation model used is RAV, vehicles follow the Krauss mobility model, there are 3 warning mode vehicles, the periodicity of messages is 1 message per second, normal message priority is AC0, the broadcast scheme applied is eMDR, and the channel bandwidth is 6 Mbps.

As expected, the warning notification time is lower when the vehicle density increases. When simulating with 400 vehicles, information reaches about 60% of the vehicles in only 1.3 s, and the propagation process is completed in 2.4 s.

Table 8 shows the percentage of blind vehicles and the number of packets received per vehicle when varying the density of vehicles. The behavior in terms of percentage of blind vehicles highly depends on this factor. This characteristic is explained because the flooding propagation of warning messages works better with higher vehicle densities. As for the number of packets received per vehicle, this number highly increases when increasing vehicle density.

**Figure 6.**
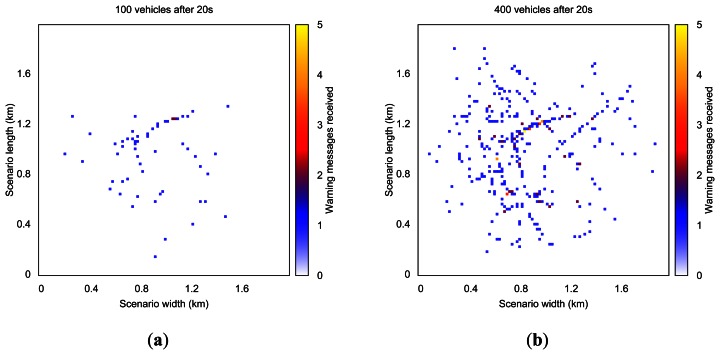
Evolution of the warning message dissemination process in the Rome scenario after 20 s, when simulating (**a**) 100 and (**b**) 400 vehicles.

Figure 6 shows the number of warning messages received in each area when simulating 100 and 400 vehicles, respectively. When only 100 vehicles are simulated the dissemination process presents a very slow progression. If the simulations include 400 vehicles, the dissemination process is able to reach a wider area of the scenario since finding appropriate rebroadcasting nodes becomes easier.

### 5.4. Evaluating the Impact of the Roadmap

This subsection presents the results obtained when varying the roadmap used. We selected scenarios from New York, San Francisco, and Rome. In Table 9 we present the main features of the chosen fragments of the cities.

Table 4 shows some of the parameters used for the simulations; the rest of parameters are the following: 200 vehicles are simulated, the radio propagation model used is RAV, vehicles follow the Krauss mobility model, there are 3 warning mode vehicles, the periodicity of messages is 1 message per second, normal message priority is AC0, the broadcast scheme applied is eMDR, and the channel bandwidth is 6 Mbps.

**Figure 7.**
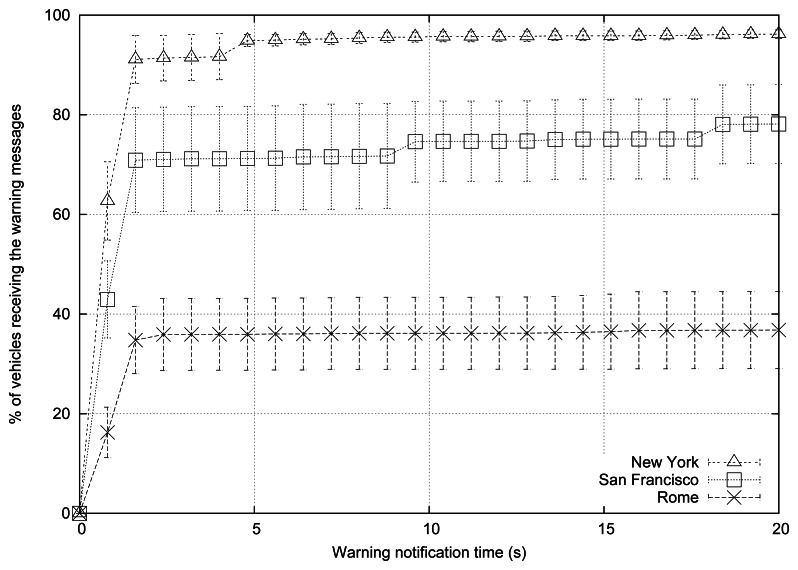
Warning notification time when varying the roadmap.

As shown, the warning notification time is lower when simulating the New York map (see Figure 7). Information reaches about 60% of the vehicles in less than 0.8 s, and propagation is completed in 5 s. When simulating the map of San Francisco, information needs more time (1.4 s) to reach the same percentage of vehicles. As for Rome, the propagation process was completed in only 2.4 s, but less than 40% of the vehicles are informed.

The behavior in terms of percentage of blind vehicles and the number of packets received also highly depends on this factor (see Table 10). In fact, when simulating New York, the percentage of blind vehicles is almost negligible, while we find 60.92% of blind vehicles when simulating Rome. Hence, when the simulated layout is more complex, the percentage of blind vehicles increases, and more time is needed to reach the same percentage of vehicles. This occurs mainly because the signal propagation is blocked by buildings. Moreover, the average number of packets received per vehicle highly differs depending on the map. Compared with New York, the number of packets received decreases considerably for San Francisco and even more for Rome since signal propagation encounters more restrictions.

**Figure 8.**
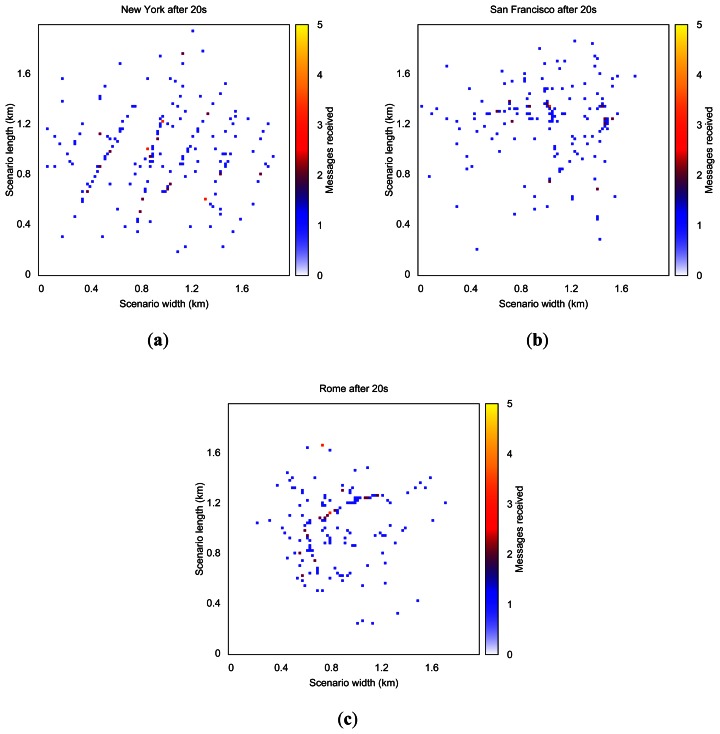
Evolution of the warning message dissemination process after 20 s, when simulating (**a**) New York; (**b**) San Francisco; and (**c**) Rome scenarios.

Figure 8 shows the number of warning messages received in each area when simulating New York, San Francisco, and Rome, respectively. As mentioned before, when simulating the New York scenario the dissemination process is able to reach a wider area since streets are longer and wider, and there are fewer junctions, so messages can be disseminated more easily.

### 5.5. Lessons Learnt and Guidelines for Future Research

The 2*^k^* factorial analysis has shown that the key factors to take into account when simulating VANETs are: (i) the radio propagation model; (ii) the density of vehicles; and (iii) the roadmap used. By evaluating the impact of each factor one by one, we confirmed the outcome of the 2*^k^* factorial analysis. We observed that the results obtained are highly affected by the selected radio propagation model, the roadmap and the density of vehicles. The propagation of warning messages works better with simpler layouts and higher vehicle densities.

Results also showed that other important factors, such as the broadcast scheme used, the channel bandwidth, and the priority and the periodicity of messages, have little impact in the warning message delivery process. Nevertheless, we believe that these parameters could be important factors in other VANET scenarios and applications, such as live video streaming services to vehicles.

Although the selected roadmap is a key factor in VANETs, the majority of VANET proposals tend to use very simplistic scenarios. We consider that the use of more realistic topologies is required in order to obtain meaningful results. However, the very large number of possible scenarios and the differences among them become a drawback when attempting to follow our strategy. Thus, in the next section we present a roadmap profile classification that will be very useful for future VANET research works by aggregating cities into a same group depending on their characteristics.

## 6. Roadmap Profile Classification

Above we have shown that the specific features of the scenarios must be taken into account to make the future proposals more representative and valid. To achieve this goal, maps from several existing cities have been tested to obtain a classification that allows future researchers to determine which scenarios to use in their simulations. In each scenario analyzed, the chosen area tries to represent the overall layout of the streets in each city, and is usually taken from downtown. We selected representative cities from Europe (Berlin, London, Milan, Moscow, Paris, Rome, Seville, Teruel, Valencia), Asia (Beijing, Hong Kong, Istanbul, Kuala Lumpur, New Delhi, Seoul, Shanghai, Taipei, Tokyo), North America (Boston, Chicago, Los Angeles, Manhattan, Mexico City, New York, San Francisco, Washington DC), South America (Buenos Aires, Montevideo, Rio de Janeiro), and Africa (Cape Town, Casablanca, El Cairo, Rabat).

**Figure 9.**
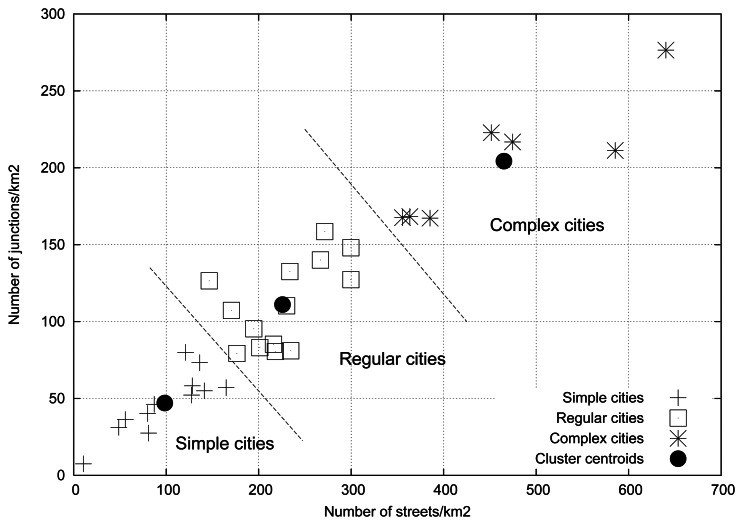
Classification of different cities based on the density of streets and junctions.

Figure 9 shows the number of streets and junctions present in a 4 km^2^ square area in these cities. In this work, each segment between two junctions is considered a street. As shown, the relationship between the number of streets and the number of junctions is almost linear, in an approximate ratio of 2 streets per junction.

Results shown in Figure 7 suggest that three different performance profiles can be identified. According to this, we used the well-known *k-means* clustering algorithm [42] with a number of clusters *k* = 3 to obtain a precise classification of the cities. By using the results of the clustering process in Figure 9, we can classify a new city according to the cluster whose centroid is the nearest (using the Euclidean distance as a measure). We can classify existing cities by their street profiles into:

- *Simple layouts*: maps with low density of streets and junctions. Usually arranged orthogonally like a Manhattan style grid. Examples of these cities are New York (USA), Moscow (Russia), Los Angeles (USA), and Seoul (South Korea).
- *Regular layouts*: maps with medium density of streets and junctions. Some cities in this group are San Francisco (USA), Madrid (Spain), Washington DC (USA) and Paris (France).
- *Complex layouts*: maps with high density of streets and junctions. Cities that belong to this group are Rome (Italy), London (UK), Valencia (Spain), and Tokyo (Japan).

Table 11 summarizes the classification process of the studied cities and shows the location of the centroid of the cluster assigned to each profile.

Previous results (in Section 5) showed that the roadmap that serves as scenario for the warning dissemination has a considerable influence on the effectiveness of the process. Moreover, we can differentiate three groups of roadmap profiles in which the propagation process is likely to behave in a similar way. Thus, we consider that researchers must carefully determine the scenarios to assess their proposals since the obtained results will be directly affected by the roadmap used. In particular, we recommend to test with at least one map for each roadmap profile to make sure that results are representative and conclusions sufficiently generic.

### 6.1. Assessing the Roadmap Profile Classification

Simulation experiments have shown that the features of each specific scenario determine the efficiency of the dissemination process. To prove how maps from the same cluster produce similar results using them as simulation scenarios, we selected three street maps in addition to those presented in Figure 2. These additional roadmaps are taken from different cities and they belong to different clusters, as shown in Table 12. The scenarios were obtained from OpenStreetMap, each one representing 4 km^2^ of square area.

**Figure 10.**
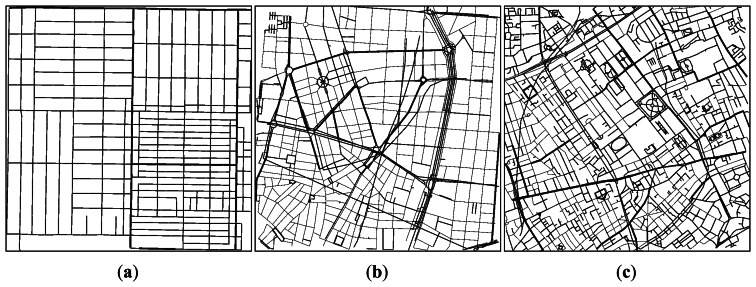
Additional scenarios used in our simulations as street graphs in SUMO: (**a**) fragment of the city of Los Angeles (USA); (**b**) fragment of the city of Madrid (Spain); and (**c**) fragment of the city of London (UK).

Figure 10(a) shows the area between Martin Luther King Boulevard and West Slauson Avenue in the city of Los Angeles (CA, USA), which belongs to the Simple layout cluster. It has a very regular street layout where the simulations should have a similar behavior compared to simulations performed using synthetic Manhattan-grid layouts. The street map around Paseo de la Castellana in the city of Madrid (Spain), shown in Figure 10(b), is classified as a Regular profile. It is an example of town with medium density of streets and junctions, arranged in a complex layout different from typical Manhattan-grid layouts. Finally, Figure 10(c) presents the area around Russell Square in the city of London (UK), which contains an extremely high density of streets and junctions, and therefore it belongs to the Complex topologies cluster. We will study warning message dissemination efficiency in these scenarios and we will compare the results with those obtained with the formerly presented roadmaps.

### 6.2. Comparison Results

**Figure 11.**
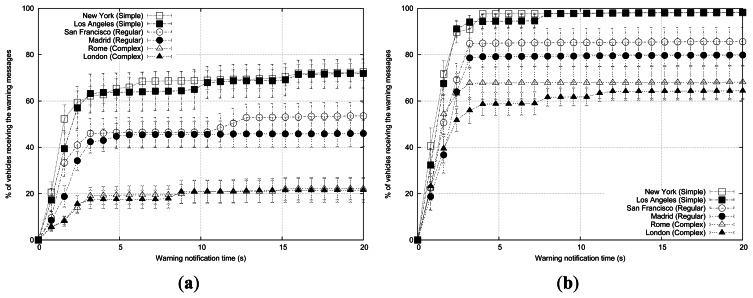
Warning notification time in different scenarios simulating (**a**) 100 vehicles and (**b**) 400 vehicles.

**Figure 12.**
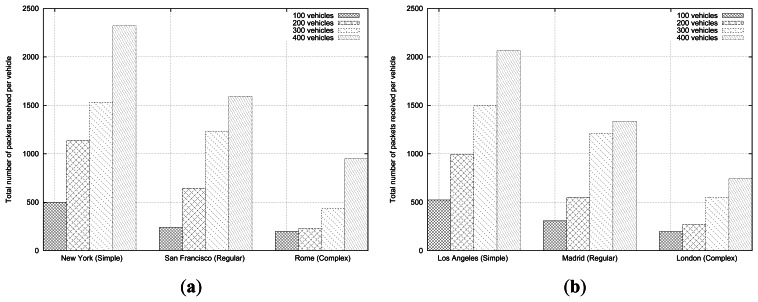
Number of messages received per vehicle simulating (**a**) the formerly presented scenarios, and (**b**) the additional street maps, under different vehicle densities.

Results in this section are obtained using the maps of New York, San Francisco and Rome from Figure 2, and also the roadmaps from Los Angeles, Madrid and London from Figure 10. There is a city from each defined cluster in these two sets of roadmaps, and we will compare warning message dissemination using these different topologies. Figures 11 and 12 show the differences in terms of both warning notification time and messages received per vehicle when varying the density of vehicles in the aforementioned city scenarios. In all these simulations we used the same base configuration: the radio propagation model used is RAV, vehicles follow the Krauss mobility model, there are 3 warning mode vehicles, the periodicity of messages is 1 message per second, normal message priority is AC0, the broadcast scheme applied is eMDR, and the channel bandwidth is 6 Mbps.

Results in Figure 11 show that the selected scenario notably affects the efficiency of the dissemination process, especially in scenarios with low vehicle density. As the density of vehicles grows, the differences become smaller but they are still noticeable. In addition, roadmaps from the same cluster present a very similar behavior in both low and high vehicle density scenarios. Topologies from the Simple layout cluster obtain the best performance in warning notification time and percentage of blind vehicles in all scenarios, since the wireless signal propagates more easily in environments with few long streets. As the layout becomes more irregular and the density of streets and junctions grows, the dissemination process develops more slowly and the number of uninformed vehicles increases.

In the six scenarios, increasing the density of vehicles yields better performance in terms of both warning notification time and percentage of blind vehicles (*i.e.*, not receiving warning messages), especially in roadmaps like Rome and London where the streets are the shortest and the most irregular, producing very poor results when there are few vehicles in the simulated scenario. Complex layout scenarios need higher vehicle densities to obtain satisfactory results in terms of warning notification time and blind vehicles.

As shown in Figure 12, topologies from the same cluster also produce a similar number of messages. For Simple roadmaps there is a sudden increment in the amount of received messages when the number of vehicles grows more than 100 vehicles, whereas the Regular ones support up to 200 vehicles, and Complex roadmaps obtain sustainable results up to 300 vehicles. Note that urban scenarios with low density of streets and junctions greatly increase the number of messages received per vehicle because of the higher number of vehicles reached by the wireless signal, thanks to the long streets forming the layout that make it easier to find vehicles in line-of-sight.

## 7. Conclusions

In this paper, we identified and described the different factors to be taken into account when simulating warning message dissemination in VANETs. Since the number of possible factors can be very large, we identified the representative factors by using the 2*^k^* factorial analysis. The purpose is to reduce the required simulation time in future research works.

The key factors affecting the delivery of warning messages were found to be the radio propagation model, the density of vehicles, and the roadmap used. Some other factors, such as the broadcast scheme used, the channel bandwidth, and the priority and the periodicity of messages, did not have a significant impact on the metrics considered in our study. We believe that the results of our analysis can save researchers' time by discarding unnecessary factors when performing simulations for VANET-related research.

Results obtained from our simulations confirmed that the selected roadmap is a crucial factor. In fact, performance parameters such as warning notification time, the percentage of blind vehicles, and the number of packets received per vehicle highly depend on it. To further reduce the scope of warning message dissemination tests made in real cities, we propose and evaluate a scenario classification based on three roadmap profiles, and consider that researchers must carefully determine the scenarios to assess their proposals, ideally picking at least one scenario for each profile type.

## Figures and Tables

**Table 1.** Experiments defined by a 2^2^ design.

**Experiment**	**A**	**B**	**y**
1	–1	–1	*y*_1_
2	1	–1	*y_2_*
3	–1	1	*y*_3_
4	1	1	*y*_4_

**Table 2.** Example of results obtained in terms of warning notification time varying two factors.

**Density of vehicles**	**Speed 10 km/h**	**Speed 80 km/h**
25 veh./km^2^	1s	0.8 s
150 veh./km^2^	0.5 s	0.4 s

**Table 3.** Sign table method of calculating the effects of the factors in a 2^2^ design.

**I**	**A**	**B**	**AB**	**y**
1	–1	–1	1	1s
1	1	–1	–1	0.5 s
1	–1	1	–1	0.8 s
1	1	1	1	0.4 s

2.7	–0.9	–0.3	0.1	Total
0.675	–0.225	–0.075	0.025	Total/4

**Table 4.** Parameters used for the simulations.

**Parameter**	**Value**
roadmap size	2,000 m × 2,000 m
downtown size	1,000 m × 1,000 m
downtown probability	0.5
downtown attraction	0.5
warning packet size	256B
normal packet size	512B
warning messages priority	AC3
MAC/PHY	802.11p
maximum transmission range	400 m

**Table 5.** Factors considered and their values.

**Factor**	**Level–1**	**Level 1**
warning vehicles (A)	3	10
density of vehicles (B)	25 vehicles/km^2^	100 vehicles/km^2^
channel bandwidth (C)	3 Mbps	6 Mbps
broadcast scheme (D)	location-based [27]	eMDR [21]
normal messages priority (E)	AC0	AC3
periodicity of messages (F)	1 packet/s	20 packets/s
mobility model (G)	Krauss modified [37]	Wagner [38]
radio propagation model (H)	Two Ray Ground	RAV [4]
roadmap (I)	New York	Rome

**Table 6.** The percentage of variation explained using the sign table method up to the combination of 2 factors. Highlighted values indicate representative variations.

**Factors**	**Variation Explained (%)**		

	**Warning Notification Time**	**% of Blind Vehicles**	**Number of Packets Received**
*A*	5.67	1.88	1.13
*B*	0.89	**8.61**	**28.55**
*C*	0.02	0.00	3.63
*D*	3.22	0.14	3.28
*E*	0.00	0.00	0.00
*F*	0.00	0.00	0.00
*G*	0.47	2.70	0.23
*H*	**20.72**	**49.87**	**36.26**
*I*	**9.35**	**14.07**	**7.92**
*AB*	0.37	1.30	0.05
*AC*	0.06	0.00	0.19
*AD*	0.77	0.03	0.59
*AE*	0.00	0.00	0.00
*AF*	0.00	0.00	0.00
*AG*	0.01	0.35	0.61
*AH*	0.09	1.87	0.29
*AI*	0.90	0.15	0.01
*BC*	0.05	0.00	2.39
*BD*	1.03	0.06	0.22
*BE*	0.00	0.00	0.00
*BF*	0.00	0.00	0.00
*BG*	**33.35**	0.42	0.56
*BH*	**14.40**	**9.09**	5.62
*BI*	1.06	5.37	4.21
*CD*	0.07	0.00	0.06
*CE*	0.00	0.00	0.00
*CF*	0.00	0.00	0.00
*CG*	0.00	0.00	0.05
*CH*	0.01	0.00	1.59
*CI*	0.03	0.00	0.51
*DE*	0.00	0.00	0.00
*DF*	0.00	0.00	0.00
*DG*	5.26	0.06	0.25
*DH*	0.07	0.13	1.16
*DI*	0.94	0.13	0.03
*EF*	0.00	0.00	0.00
*EG*	0.00	0.00	0.00
*EH*	0.00	0.00	0.00
*EI*	0.00	0.00	0.00
*FG*	0.00	0.00	0.00
*FH*	0.00	0.00	0.00
*FI*	0.00	0.00	0.00
*GH*	0.25	2.60	0.00
*GI*	0.94	1.17	0.60

**Table 7.** Blind vehicles and packets received per vehicle when varying the Radio Propagation Model.

**RPM**	**% of Blind Vehicles**	**Packets Received**
TRG	0%	3,417.10
Nakagami	0.1%	1,291.10
RAV	60.92%	229.07

**Table 8.** Blind vehicles and packets received per vehicle when varying the density of vehicles.

**Vehicles**	**% of Blind Vehicles**	**Packets Received**
100	76.63%	197.37
200	60.92%	229.07
300	36.40%	432.60
400	21.01%	949.40

**Table 9.** Main features of the selected maps.

**Selected city map**	**New York (USA)**	**San Francisco (USA)**	**Rome (Italy)**
**Streets/km^2^**	175	428	695
**Junctions/km^2^**	125	205	298
**Avg. street length**	122.55 m	72.71 m	45.89 m
**Avg. lanes/street**	1.57	1.17	1.06

**Table 10.** Blind vehicles and packets received per vehicle when varying the roadmap.

**Roadmap**	**% of Blind Vehicles**	**Packets Received**
New York	2.92%	1,542.07
San Francisco	20.55%	885.13
Rome	60.92%	229.07

**Table 11.** Roadmap Profiles Classification.

**Roadmap Profile**	**Street and Junction Density**	**Cluster Centroid**	

		**Streets/km^2^**	**Junctions/km^2^**
Simple	Low	98.5	47.06
Regular	Medium	225.72	111.04
Complex	High	465.14	204.32

**Table 12.** Main features of the additional maps.

**Selected City Map**	**Los Angeles (USA)**	**Madrid (Spain)**	**London (UK)**
**Streets/km^2^**	263	479	878
**Junctions/km^2^**	77	284	408
**Avg. street length**	111.58 m	67.23 m	45.38 m
**Avg. lanes/street**	1.45	1.26	1.15

**Profile cluster**	Simple	Regular	Complex

## References

[1] Galaviz-Mosqueda G.A., Aquino-Santos R., Villarreal-Reyes S., Rivera-Rodriguez R., Villaseñor Gonzalez L., Edwards A. (2012). Reliable freestanding position-based routing in highway scenarios. Sensors.

[2] Gramaglia M., Bernardos C.J., Calderon M. (2013). Virtual induction loops based on cooperative vehicular communications. Sensors.

[3] Rahim A., Khan Z., Muhaya F.T.B., Sher M., Kim T.H. (2010). Sensor based framework for secure multimedia communication in VANET. Sensors.

[4] Martinez F.J., Fogue M., Toh C.K., Cano J.C., Calafate C.T., Manzoni P. (2012). Computer simulations of VANETs using realistic city topologies. Wirel. Pers. Commun..

[5] IEEE 802.11 Working Group (2010). IEEE Standard for Information Technology–Telecommunications and Information Exchange between Systems–Local and Metropolitan Area Networks–Specific Requirements–Part 11: Wireless LAN Medium Access Control (MAC) and Physical Layer (PHY) Specifications Amendment 6: Wireless Access in Vehicular Environments.

[6] Jain R. (1991). The Art of Computer Systems Performance Analysis: Techniques for Experimental Design, Measurement, Simulation, and Modelling.

[7] Zuo J., Wang Y., Liu Y., Zhang Y. Performance Evaluation of Routing Protocol in VANET with Vehicle-Node Density.

[8] Giordano E., Frank R., Ghosh A., Pau G., Gerla M. Two Ray or not Two Ray this is the Price to Pay.

[9] Khorashadi B., Chen A., Ghosal D., Chuah C.N., Zhang M. Impact of Transmission Power on the Performance of UDP in Vehicular *Ad Hoc* Networks.

[10] Cenerario N., Delot T., Ilarri S. (2011). A content-based dissemination protocol for VANETs: Exploiting the encounter probability. IEEE Trans. Intell. Transp. Syst..

[11] Sahoo J., Wu E.K., Sahu P., Gerla M. (2011). Binary-partition-assisted MAC-layer broadcast for emergency message dissemination in VANETs. IEEE Trans. Intell. Transp. Syst..

[12] Costa P., Frey D., Migliavacca M., Mottola L. Towards Lightweight Information Dissemination in Inter-Vehicular Networks.

[13] Viriyasitavat W., Bai F., Tonguz O. UV-CAST: An Urban Vehicular Broadcast Protocol.

[14] Liu C., Chigan C. (2012). RPB-MD: Providing robust message dissemination for vehicular Ad Hoc networks. Ad Hoc Netw..

[15] Gupta R.A., Agarwal A.K., Chow M.Y., Wang W. Performance Assessment of Data and Time-Sensitive Wireless Distributed Networked-Control-Systems in Presence of Information Security.

[16] Liu C., MacGregor M.H., Harms J. (2008). Improving Multipath Routing Performance in WSNs by Tuning IEEE 802.11 Parameters.

[17] Vaz de Melo P.O., da Cunha F.D., Almeida J.M., Loureiro A.A., Mini R.A. (2008). The Problem of Cooperation among Different Wireless Sensor Networks.

[18] Perkins D., Hughes H., Owen C. Factors Affecting the Performance of *Ad Hoc* Networks.

[19] Perkins D., Hughes H. (2002). Investigating the performance of TCP in mobile *Ad Hoc* networks. Comput. Commun..

[20] Fogue M., Garrido P., Martinez F.J., Cano J.C., Calafate C.T., Manzoni P. Analysis of the Most Representative Factors Affecting Warning Message Dissemination in VANETs under Real Roadmaps.

[21] Fogue M., Garrido P., Martinez F.J., Cano J.C., Calafate C.T., Manzoni P. (2012). Evaluating the impact of a novel message dissemination scheme for Vehicular Networks using real maps. Transp. Res. Part C Emerg. Technol..

[22] Sanguesa J.A., Fogue M., Garrido P., Martinez F.J., Cano J.C., Calafate C.T., Manzoni P. (2013). An infrastructureless approach to estimate vehicular density in urban environments. Sensors.

[23] Jiang D., Chen Q., Delgrossi L. Communication Density: A Channel Load Metric for Vehicular Communications Research.

[24] Eichler S. Performance Evaluation of the IEEE 802.11p WAVE Communication Standard.

[25] Jiang D., Chen Q., Delgrossi L. (2008). Optimal Data Rate Selection for Vehicle Safety Communications.

[26] Liu C., Chigan C. (2012). RPB-MD: Providing robust message dissemination for vehicular *Ad Hoc* networks. Ad Hoc Netw..

[27] Tseng Y.C., Ni S.Y., Chen Y.S., Sheu J.P. (2002). The broadcast storm problem in a mobile *Ad Hoc* network. Wirel. Netw..

[28] Wisitpongphan N., Tonguz O., Parikh J., Mudalige P., Bai F., Sadekar V. (2007). Broadcast storm mitigation techniques in vehicular *Ad Hoc* networks. IEEE Wirel. Commun..

[29] Suriyapaibonwattana K., Pornavalai C. An Effective Safety Alert Broadcast Algorithm for VANET.

[30] Alasmary W., Zhuang W. (2012). Mobility impact in IEEE 802.11p infrastructureless vehicular networks. Ad Hoc Netw..

[31] Harri J., Filali F., Bonnet C. (2009). Mobility models for vehicular *Ad Hoc* networks: A survey and taxonomy. IEEE Commun. Surv. Tutor..

[32] Krajzewicz D., Rossel C. Simulation of Urban MObility (SUMO). http://sumo.sourceforge.net.

[33] OpenStreetMap, Collaborative Project to Create a Free Editable Map of the World, 2011.

[34] TIGER, Topologically Integrated Geographic Encoding and Referencing, 2011.

[35] Martinez F.J., Cano J.C., Calafate C.T., Manzoni P. CityMob: A Mobility Model Pattern Generator for VANETs.

[36] Krauss S., Wagner P., Gawron C. (1997). Metastable states in a microscopic model of traffic flow. Phys. Rev. E..

[37] Krajzewicz D., Hertkorn G., Rossel C., Wagner P. SUMO (Simulation of Urban MObility)—An Open-Source Traffic Simulation.

[38] Wagner P. (2006). How human drivers control their vehicle. Eur. Phys. J. B..

[39] Martinez F.J., Toh C.K., Cano J.C., Calafate C.T., Manzoni P. Realistic Radio Propagation Models (RPMs) for VANET Simulations.

[40] Fall K., Varadhan K. ns Notes and Documents. The VINT Project. UC Berkeley, LBL, USC/ISI, and Xerox PARC, 2000.

[41] Fogue M., Garrido P., Martinez F.J., Cano J.C., Calafate C.T., Manzoni P. A Realistic Simulation Framework for Vehicular Networks.

[42] MacQueen J.B., Cam L.M.L., Neyman J. (1967). Some Methods for Classification and Analysis of MultiVariate Observations.

